# Supplementary material for: Pyruvate Dehydrogenase Kinase 1 inhibition mediated oxidative phosphorylation enhancement in cartilage promotes osteoarthritis progression
Source: BMC Musculoskelet Disord. 2023 Jul 20;24:597. doi: 10.1186/s12891-023-06585-6 (PMC10357736; doi:10.1186/s12891-023-06585-6)
Supplement: Supplementary file 1 — Additional file 1. [file 12891_2023_6585_MOESM1_ESM.zip › MF.Gsea.1653627316242/GOMF_GLUTATHIONE_TRANSFERASE_ACTIVITY.html]

Details for gene set GOMF\_GLUTATHIONE\_TRANSFERASE\_ACTIVITY[GSEA]

|  || Dataset | 55457\_collapsed\_to\_symbols.cls.cls #OA\_versus\_normal.cls.cls #OA\_versus\_normal\_repos |
| Phenotype | cls.cls#OA\_versus\_normal\_repos |
| Upregulated in class | OA |
| GeneSet | GOMF\_GLUTATHIONE\_TRANSFERASE\_ACTIVITY |
| Enrichment Score (ES) | 0.6474633 |
| Normalized Enrichment Score (NES) | 2.12495 |
| Nominal p-value | 0.0 |
| FDR q-value | 0.0022442145 |
| FWER p-Value | 0.02 |
Table: GSEA Results Summary

  

Fig 1: Enrichment plot: GOMF\_GLUTATHIONE\_TRANSFERASE\_ACTIVITY      
 Profile of the Running ES Score & Positions of GeneSet Members on the Rank Ordered List

  

| SYMBOL | TITLE | RANK IN GENE LIST | RANK METRIC SCORE | RUNNING ES | CORE ENRICHMENT || 1 | GSTA4 | glutathione S-transferase alpha 4 [Source:HGNC Symbol;Acc:HGNC:4629] | 216 | 0.808 | 0.0752 | Yes |
| 2 | LTC4S | leukotriene C4 synthase [Source:HGNC Symbol;Acc:HGNC:6719] | 223 | 0.804 | 0.1663 | Yes |
| 3 | GSTK1 | glutathione S-transferase kappa 1 [Source:HGNC Symbol;Acc:HGNC:16906] | 490 | 0.662 | 0.2211 | Yes |
| 4 | HPGDS | hematopoietic prostaglandin D synthase [Source:HGNC Symbol;Acc:HGNC:17890] | 605 | 0.622 | 0.2831 | Yes |
| 5 | MGST3 | microsomal glutathione S-transferase 3 [Source:HGNC Symbol;Acc:HGNC:7064] | 610 | 0.620 | 0.3534 | Yes |
| 6 | GSTZ1 | glutathione S-transferase zeta 1 [Source:HGNC Symbol;Acc:HGNC:4643] | 974 | 0.523 | 0.3848 | Yes |
| 7 | GSTM3 | glutathione S-transferase mu 3 [Source:HGNC Symbol;Acc:HGNC:4635] | 1025 | 0.512 | 0.4393 | Yes |
| 8 | GSTT1 | glutathione S-transferase theta 1 [Source:HGNC Symbol;Acc:HGNC:4641] | 1129 | 0.491 | 0.4871 | Yes |
| 9 | CLIC2 | chloride intracellular channel 2 [Source:HGNC Symbol;Acc:HGNC:2063] | 1214 | 0.473 | 0.5345 | Yes |
| 10 | GSTP1 | glutathione S-transferase pi 1 [Source:HGNC Symbol;Acc:HGNC:4638] | 1260 | 0.465 | 0.5840 | Yes |
| 11 | LANCL1 | LanC like 1 [Source:HGNC Symbol;Acc:HGNC:6508] | 1507 | 0.428 | 0.6136 | Yes |
| 12 | ALOX5AP | arachidonate 5-lipoxygenase activating protein [Source:HGNC Symbol;Acc:HGNC:436] | 1665 | 0.404 | 0.6475 | Yes |
| 13 | MGST2 | microsomal glutathione S-transferase 2 [Source:HGNC Symbol;Acc:HGNC:7063] | 2583 | 0.292 | 0.6096 | No |
| 14 | GSTM5 | glutathione S-transferase mu 5 [Source:HGNC Symbol;Acc:HGNC:4637] | 3123 | 0.238 | 0.5948 | No |
| 15 | GSTM4 | glutathione S-transferase mu 4 [Source:HGNC Symbol;Acc:HGNC:4636] | 3144 | 0.235 | 0.6200 | No |
| 16 | GSTA3 | glutathione S-transferase alpha 3 [Source:HGNC Symbol;Acc:HGNC:4628] | 3807 | 0.176 | 0.5886 | No |
| 17 | PTGES | prostaglandin E synthase [Source:HGNC Symbol;Acc:HGNC:9599] | 4399 | 0.121 | 0.5565 | No |
| 18 | GSTM1 | glutathione S-transferase mu 1 [Source:HGNC Symbol;Acc:HGNC:4632] | 5848 | 0.005 | 0.4447 | No |
| 19 | GSTO1 | glutathione S-transferase omega 1 [Source:HGNC Symbol;Acc:HGNC:13312] | 6275 | -0.025 | 0.4145 | No |
| 20 | GSTT2 | glutathione S-transferase theta 2 (gene/pseudogene) [Source:HGNC Symbol;Acc:HGNC:4642] | 7666 | -0.132 | 0.3216 | No |
| 21 | GSTT4 | glutathione S-transferase theta 4 [Source:HGNC Symbol;Acc:HGNC:26930] | 8923 | -0.226 | 0.2498 | No |
| 22 | GSTA1 | glutathione S-transferase alpha 1 [Source:HGNC Symbol;Acc:HGNC:4626] | 11608 | -0.517 | 0.1003 | No |
Table: GSEA details [plain text format]

  

Fig 2: GOMF\_GLUTATHIONE\_TRANSFERASE\_ACTIVITY      
 Blue-Pink O' Gram in the Space of the Analyzed GeneSet

  

Fig 3: GOMF\_GLUTATHIONE\_TRANSFERASE\_ACTIVITY: Random ES distribution      
 Gene set null distribution of ES for **GOMF\_GLUTATHIONE\_TRANSFERASE\_ACTIVITY**
